# Incidence, characteristics and long-term outcomes of patients with diabetic ketoacidosis: a prospective prognosis cohort study in an emergency department

**DOI:** 10.1590/1516-3180.2020.0285.R1.21102020

**Published:** 2021-02-24

**Authors:** Rachel Teixeira Leal Nunes, Carolina Frade Magalhães Girardin Pimentel Mota, Paulo Ricardo Gessolo Lins, Fernanda Salles Reis, Thais Castanheira de Freitas Resende, Ludmila de Andrade Barberino, Pedro Henrique Luiz da Silva, Aecio Flavio Teixeira de Gois

**Affiliations:** I MD, MSc. Physician, Department of Medicine, Universidade Federal de São Paulo (UNIFESP), São Paulo (SP), Brazil.; II MD, PhD. Adjunct Professor, Department of Medicine, Universidade Federal de São Paulo (UNIFESP), São Paulo (SP), Brazil.; III MD. Doctoral Student, Department of Medicine, Universidade Federal de São Paulo (UNIFESP), São Paulo (SP), Brazil.; IV MD, MSc. Doctoral Student, Department of Medicine, Universidade Federal de São Paulo (UNIFESP), São Paulo (SP), Brazil.; V MD, Doctoral Student, Endocrinology Division, Universidade de São Paulo (USP), São Paulo (SP), Brazil.; VI MD. Physician, Cardiology Division, Universidade de São Paulo (USP), São Paulo (SP), Brazil.; VII MD. Master’s Student, Department of Medicine, Universidade Federal de São Paulo (UNIFESP), São Paulo (SP), Brazil.; VIII MD, PhD, Adjunct Professor, Department of Medicine, Universidade Federal de São Paulo (UNIFESP), São Paulo (SP), Brazil.

**Keywords:** Diabetic ketoacidosis, Diabetes complications, Emergency service, hospital, Mortality, Complications of diabetes mellitus, Emergency department, Mortalities

## Abstract

**BACKGROUND:**

Diabetic ketoacidosis is the most frequent hyperglycemic complication in the evolution of diabetes mellitus. Common precipitating factors include newly diagnosed diabetes mellitus, noncompliance with therapy and infections. However, few studies have been conducted in Brazil and none were prospective in design.

**OBJECTIVE:**

To describe the incidence, clinical and laboratory characteristics and precipitating factors of diabetic ketoacidosis among emergency department patients in a tertiary-level teaching hospital in Brazil. We also aimed to identify immediate and long-term mortality within two years.

**DESIGN AND SETTING:**

Prospective prognosis cohort study conduct at a tertiary-level teaching hospital in São Paulo, Brazil.

**METHODS:**

All patients > 12 years old presenting diabetic ketoacidosis who were admitted to the emergency department from June 2015 to May 2016 were invited to participate.

**RESULTS:**

The incidence of diabetic ketoacidosis per 1,000 admissions was 8.7. Treatment noncompliance and infection were the most common causes of diabetic ketoacidosis. The immediate mortality rate was 5.8%, while the six-month, one-year and two-year mortality rates were 9.6%, 13.5% and 19.2%, respectively. Death occurring within two years was associated with age, type 2 diabetes, hypoalbuminemia, infection at presentation and higher sequential organ failure assessment (SOFA) score at admission.

**CONCLUSIONS:**

Diabetic ketoacidosis among patients presenting to the emergency department was relatively frequent in our hospital. Treatment noncompliance and infection were major precipitating factors and presence of diabetic ketoacidosis was associated with immediate and long-term risk of death.

## INTRODUCTION

Diabetic ketoacidosis (DKA) is the most frequent hyperglycemic complication in the evolution of diabetes mellitus (DM).^1^ It represents a state of metabolic stress and is characterized by a pathological imbalance of insulin deficiency and counterregulatory hormones (glucagon, catecholamines, cortisol and growth hormone), thus forming a triad of marked hyperglycemia, ketosis and metabolic acidosis.^2^^,^^3^

Despite the situation of hyperglycemia, the lack of insulin in the setting of elevated counter-regulatory hormones means that glucose is not properly used as energy and ketone production exceeds peripheral utilization. Ultimately, this leads to development of DKA, given that the increase in ketoacid production is so great that metabolic acidosis occurs. Patients with DKA are usually admitted to the emergency department presenting with hyperglycemia associated with polyuria, polydipsia and significant dehydration.^4^^,^^5^

Successful therapy requires correction of hypovolemia, hyperglycemia, metabolic acidosis and electrolyte imbalances. Frequent monitoring is also necessary, in order to restore the patient to a normal metabolic state.^2^^,^^3^ Crucially, the underlying cause of the ketoacidosis needs to be identified and treated, because DKA-related mortality is usually a consequence of the underlying illness.^6^ The common precipitating factors of DKA include newly-diagnosed DM, patient noncompliance with therapy and infections.^7^^,^^8^^,^^9^^,^^10^

In most studies, the mortality rate attributed to DKA has ranged from 1 to 10%.^2^^,^^3^^,^^4^ Despite the substantial literature base now available regarding DKA, few studies have yet evaluated adult DKA admissions in Brazil.^11^^,^^12^ There is thus a lack of proper information on the clinical characteristics, treatment and long-term outcomes of DKA. 

## OBJECTIVE

Therefore, we proposed and conducted a prospective prognosis cohort study to describe the incidence, clinical and laboratory findings, precipitating factors and inpatient and long-term mortality rates over a two-year period, among DKA patients admitted to the emergency department of a teaching hospital in Brazil.

## METHODS 

### Ethics

This study was approved by the Health Research Ethics Board (protocol number: 48219215.1.0000.5505; date: July 24, 2015) at the Federal University of São Paulo (Universidade Federal de São Paulo, UNIFESP. All patients enrolled in this study provided written informed consent.

### Design, setting and population

This was a prospective prognosis cohort study. All patients older than 12 years of age with a primary diagnosis of DKA who were admitted to the emergency department of the UNIFESP Teaching Hospital, in São Paulo, Brazil, between June 1, 2015, and May 31, 2016, were invited to participate. Potential cases were tracked by our study group in daily visits to the emergency department. The following patients were excluded from the study: patients presenting a hyperosmolar hyperglycemic state; women with previously known pregnancy; and patients with end-stage chronic kidney disease (CKD). Patients with end-stage CKD were excluded because the serum ketonemia test was not available at the hospital during the study period, and so the primary cause of the metabolic acidosis (i.e. whether it was renal or ketoacidosis) could not be defined. All decisions regarding patient admission and intensity of treatment were made at the discretion of the emergency department physicians, without any interference from the study group.

### Outcomes

The primary outcome of interest was the incidence of DKA among emergency department admissions. This was defined as a proportion (number of cases per number of admissions during the study period) and as the number of cases per 1,000 emergency department admissions. The secondary outcomes that were determined were the clinical characteristics and laboratory findings at admission; precipitating factors; short-term mortality rate (emergency department and hospital); long-term mortality rates (six-month, one-year and two-year); sequential organ failure assessment (SOFA) score at admission; and Acute Physiology and Chronic Health Evaluation (APACHE-II) score at admission, and its relationship with mortality rates.

### Operational definitions

The criteria used were adapted from those of the American Diabetes Association (ADA), which defines DKA as serum glucose of more than 13.9 mmol/l, moderate ketonuria or ketonemia, arterial pH less than 7.30 and serum bicarbonate less than 15 mmol/l.^2^^-^^3^ DKA severity was evaluated through an adaptation of the ADA grading criteria for severity.^2^^-^^3^ Treatment noncompliance was defined from documentation in the medical records showing non-adherence to the prescribed therapy, as a contributory factor for DKA. 

### Data sources

Data were captured from the patients’ medical records and details that were missing from the records were obtained from the patients themselves by our team, using a report form designed specifically for this study. The data collected included patients’ demographics, course of hospitalization and outcome measurements. The variables included age, sex, duration of DM, type of DM, prehospital medications, chronic kidney disease, comorbidities, precipitation factors, illness severity measurement (APACHE II), organ failure measurement (SOFA score), laboratory findings on admission, length of hospital stay and DKA outcome (discharge or death). The precipitating factors for DKA were defined by the emergency physician. 

A follow-up of up to two years was made through tracking administrative data in the hospital, to find any occurrences of outpatient visits, readmission or telephone contact.

### Statistical analysis 

All the analyses were performed using the SPSS statistical software, version 24.0 (IBM Corp., Armonk, NY, USA). For the descriptive analysis, clinical variables and univariate comparisons between variables were reported as means with standard deviations (SDs) for normally distributed or nearly-normally distributed variables, and as medians plus interquartile ranges (IQRs) for non-normally distributed continuous variables, using the Kolmogorov-Smirnov test for normality; and as frequencies and percentages for categorical variables. In order to compare immediate mortality, total mortality and other categorical parameters with continuous variables, the t test and Mann-Whitney test were used. To compare categorical parameters, the Fisher exact and chi-square tests were used. In comparing mortality with the type of diabetes and associated comorbidities, we used the likelihood ratio. Correlation inferences were made through Pearson and Spearman correlation analyses. P < 0.05 was considered statistically significant for all comparisons. 

### Patient and public involvement

We did not directly include patient and public involvement (PPI) in this study, but the database used in this study was developed with PPI and was updated by a committee that included patient representatives.

## RESULTS

**Figure 1.**
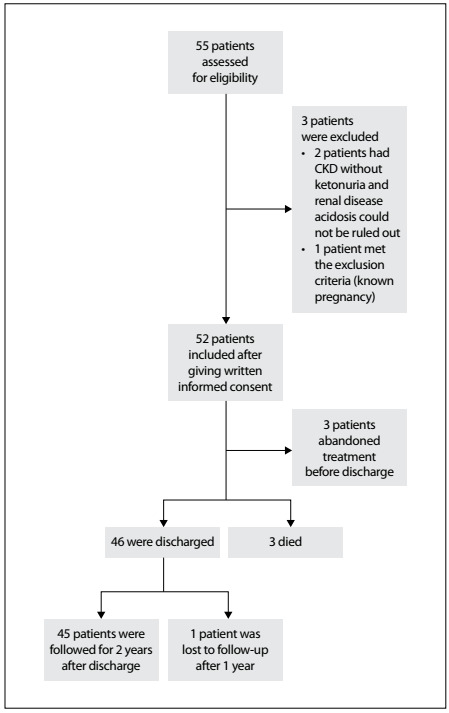
Subjects, eligibility, exclusion and follow-up.

During the study period, a total of 55 consecutive patients were admitted to the emergency department with a primary diagnosis of DKA. However, two patients were excluded from further analysis because they had end-stage chronic kidney disease and another patient was also excluded due to known pregnancy status **(**Figure 1).

The total number of admissions to the emergency department during that period was 5,943 patients. Thus, DKA accounted for 0.87% of all admissions (95% confidence interval, CI: 0.68-1.17), i.e. an estimated incidence density of 8.7 per 1,000 admissions. 

During the study period, three patients failed proceed with the care provided, despite the medical team’s advice, but they were still accounted for in the follow-up. During the two-year follow-up, only one patient was lost (Figure 1).

The initial clinical characteristics and laboratory findings of the DKA patients are presented in Table 1. Newly diagnosed DM was found in eight patients (15.4%), and 34 (65.4%) were type-1 diabetics. Considering the ADA criteria for DKA severity, 37 patients (71%) met the criteria for severe DKA, 11 (21%) had moderate DKA and 4 (8%) had mild DKA. However, only 24 (46.1%) were transferred to an intensive care unit and most patients were treated in the emergency department and then discharged after resolution, or were transferred to a ward.

**Table 1.** Demographic, clinical and laboratory characteristics of DKA patients at admission

Variable	All patients (n = 52)
Age in years, median (IQR)	28 (20-44)
Male, n (%)	23 (44)
Duration of diabetes mellitus in years, median (IQR)	10 (2.25-16.75)
Type of diabetes mellitus, n (%)	
Type 1 diabetes mellitus	34 (65.4)
Type 2 diabetes mellitus	10 (19.2)
New diabetes mellitus diagnosis	8 (15.4)
Medication use before DKA, n (%)	
Insulin use	37 (71.2)
OHA use	7 (13.5)
No treatment	8 (15.3)
Drug abuse, n (%)	4 (7.69)
Psychiatric disease, n (%)	6 (11.53)
Malignant neoplasia, n (%)	2 (3.8)
Serum glucose, mg/dl, median (IQR)	466 (393-639)
Arterial pH, mean ± SD	7.18 ± 0.15
Serum bicarbonate, millimole, mean ± SD	7.5 ± 4.8
Excess base, millimole, mean ± SD	-18 ± 6,2
Arterial lactate, millimole, mean ± SD	2.16 ± 1.12
Serum anion gap, millimole, mean ± SD	26.68 ± 8.65
Serum albumin, g/dl, median (IQR)	4.00 (3.92-4.30)
Calculated serum osmolality, mOsm/kg, median (IQR)	290 (283-304)
Serum sodium, millimole, mean ± SD	131.31 ± 5.87
Serum potassium, millimole, mean ± SD	4.95 ± 1.06
Serum chloride, millimole, mean ± SD	94.88 ± 9.5
Admission creatinine, mg/dl, median (IQR)	1,20 (0.98-1.62)
Admission urea, mg/dl, mean ± SD	50.42 ± 30.13
eGFR, ml/min/1.73 m^2^, mean ± SD	67.90 ± 33.7
History of known CKD, n (%)	5 (9.6)
Total bilirubin, mg/dl, mean ± SD	0.55 ± 0.31
Length of hospital stay in days, median (IQR)	5 (4.00-8.25)
APACHE II, points, mean ± SD	11.8 ± 6.1
SOFA, points, median (IQR)	1.5 (0-3.25)
DKA classification, n (%)	
Mild	4 (7.7)
Moderate	11 (21.2)
Severe	37 (71.1)

DKA = diabetic ketoacidosis; IQR = interquartile range; SD = standard deviation; eGFR = estimated glomerular filtration rate; CKD = chronic kidney disease; APACHE II = Acute Physiology and Chronic Health Evaluation; SOFA = sequential organ failure assessment; OHA = oral hypoglycemic agents.

**Figure 2.**
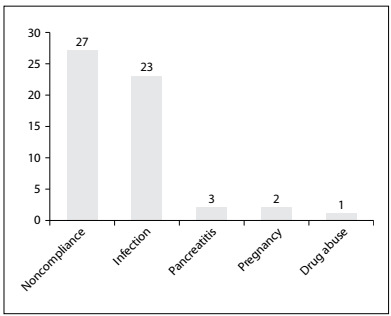
Precipitant factors in diabetic ketoacidosis. More than one cause may apply for each patient.

**Figure 3.**
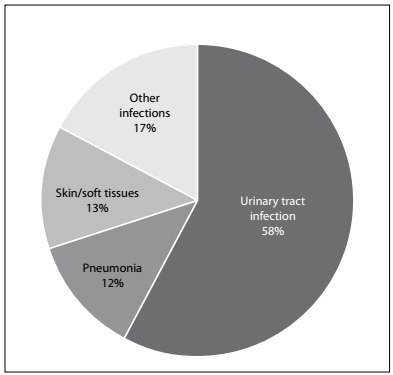
Causes of infection in patients with diabetic ketoacidosis.

The most frequent precipitating factors for DKA in our study were poor compliance with treatment and infection (Figure 2). Urinary tract infection was most common (61%), followed by pneumonia and skin or soft tissue infection. Other conditions that precipitated DKA included acute pancreatitis (3.8%), pregnancy (3.8%) and drug abuse (1.9%) (Figure 3).

The immediate mortality rate was 5.8%, and no patient was readmitted to our emergency department within a 30-day period. The six-month, one-year and two-year overall mortality rates were respectively 9.6%, 13.5% and 19.2%. The SOFA and APACHE scores did not show any correlation with immediate mortality (Table 2), although higher SOFA scores at admission were associated with long-term mortality risk. In addition to higher SOFA score, older age, type-2 DM, lower serum albumin level and presentation of infection as a precipitating factor gave rise to significant higher risk of mortality over the long term (Table 3). No other characteristics showed significant correlations with either immediate or long-term mortality.

**Table 2.** Clinical characteristics and outcomes of DKA patients according to in-hospital mortality after DKA admission

	All patients (n = 52)	In-hospital mortality		P-value
		Alive (n = 49)	Dead (n = 3)	
Age, years, median (IQR)	28 (20-4 4)	28 (20-41)	63 (42-68)	0.12^a^
Sex				0.08^b^
Male, n (%)	23 44.2)	20	3	
DM diagnosis, n				0.14^b^
New DM diagnosis	8	8	0	
DM1	35	34	1	
DM2	9	7	2	
APACHE II, points, mean ± SD	11.8 ± 6.1	12.02 ± 6.44	17 ± 11.53	0.21^a^
SOFA score, points, median (IQR)	1.5 (0-3.25)	1 (0-3)	4 (1-4 )	0.23^a^
No. of comorbidities, n (%)				0.25^b^
None	21	20	1	
1	13	14	0	
2 or more	16	14	2	
Infection				0.08^b^
No	29	29	0	
Yes	22	20	3	
DKA severity, n (%)				1.00^b^
Mild/moderate	15	14	1	
Severe	36	35	2	
pH, mean ± SD	7.18 ± 0.15	7.17 ± 0.15	7.33 ± 0.07	0.07 ^a^
Excess base, mmol/l, mean ± SD	-18 ± 6.2	-19.1 ± 6.13	-12.1 ± 3.35	0.28^a^
Serum potassium, millimole, mean ± SD	4.95 ± 1.06	5.01 ± 1.04	3.93 ± 0.86	0.15^a^
Serum anion gap, millimole, mean ± SD	26.68 ± 8.65	26.92 ± 8.71	22.83 ± 7.95	0.47^a^
Serum glucose, mg/dl, median (IQR)	466 (393-639)	459 (391-648)	558 (423-640)	0.70^a^
Serum albumin, g/dl, median (IQR)	4.00 (3.92-4.30)	4.00 (4.00-4.35)	3.50 (2.30-4.00)	0.06^a^
Osmolality, mOsm/kg, median (IQR)	290 (283-304)	290 (284-304)	300 (283-304)	0.52^a^
Lactate, mmol/l, mean ± SD	2.16 ± 1.12	2.18 ± 1.14	1.88 ± 0.83	0.66^a^
Lactate, n (%)				1.00^b^
< 2 mmol/l	28	26	2	
≥ 2 mmol/l	24	23	1	
Lactate, n (%)				1.00^b^
< 4 mmol/l	48	46	3	
≥ 4 mmol/l	3	3	0	
Duration of DM, years, mean ± SD	10.54 ± 9.66	10.23 ± 9.82	15.67 ± 5.13	0.13^a^
Length of stay, days, median (IQR)	5 (4.00-8.25)	5 (4.00-8.00)	21 (4.00-8.00)	0.12^a^

^a^Mann-Whitney U or paired t test; ^b^Fisher exact test or likelihood ratio test; DKA = diabetic ketoacidosis; IQR = interquartile range; DM = diabetes mellitus; SD = standard deviation; SOFA = sequential organ failure assessment.

**Table 3.** Clinical characteristics and outcomes of DKA patients according to end-of-follow-up (two-year) mortality after DKA admission

	All patients	End-of-follow-up (two-year) mortality		P-value
	(n = 51)	Alive (n = 41)	Dead (n = 10)	
Age, years, median (IQR)	28 (20-44)	23 (20-38)	49 (28-67)	≤ 0.001^a^
Sex				0.31^b^
Male, n (%)	23 (44.2)	17 (73.9)	6 (26.1)	
DM diagnosis, n				≤ 0.001^b^
New DM diagnosis	8	8	0	
DM1	34	29	5	
DM2	10	5	5	
APACHE II, points, mean ± SD	12.31 ± 6.76	11.93 ± 6.05	12.0 ± 7.11	0.97^a^
SOFA score, points, median (IQR)	1.5 (0-3.25)	1 (0-2)	4 (2.5-6.5)	≤ 0.001^a^
No. of comorbidities, n (%)				0.001^b^
None	22	21	1	
1	13	12	1	
2 or more	16	8	8	
Infection				≤ 0.01^b^
No	29	27 (93.1%)	2 (6.9%)	
Yes	22	14 (63.6%)	8 (36.4%)	
DKA severity, n (%)				0.13^b^
Mild/moderate	15	10 (66.7)	5 (33.3)	
Severe	36	31 (86.1)	5 (13.9)	
pH, mean ± SD	7.18 ± 0.15	7.16 ± 0.13	7.24 ± 0.22	0.32^a^
Excess base, millimole, mean ± SD	-18 ± 6.2	-19.5 ± 5.8	-21.2 ± 7.4	0.13^a^
Serum anion gap, millimole, mean ± SD	26.68 ± 8.65	27.44 ± 8.40	23.29 ± 9.50	0.76^a^
Serum potassium, millimole, mean ± SD	4.95 ± 1.06	4.98 ± 0.99	4.90 ± 1.39	0.22^a^
Osmolality, mOsm/kg, median (IQR)	290 (283-304)	290 (286-302)	296 (282-325)	0.56^a^
Serum glucose, mg/dl, median (IQR)	466 (393-639)	459 (383-648)	548 (395-687)	0.53^a^
Serum albumin, g/dl, median (IQR)	4 (3.9-4.3)	4 (4-4.6)	3.35 (3.0-3.62)	< 0.001^a^
Lactate, mmol/l, mean ± SD	2.16 ± 1.12	2.13 ± 1.09	2.36 ± 1.39	0.63^a^
Lactate, n (%)				1.00^b^
< 2 mmol/l	27	22 (82)	5 (19)	
≥ 2 mmol/l	24	19 (80)	5 (20)	
Lactate, n (%)				0.48^b^
< 4 mmol/l	48	39 (81.3)	9 (18.8)	
≥ 4 mmol/l	3	2 (66.7)	1 (33.3)	
Duration of DM, years, mean ± SD	10.69 ± 9.7	10.17 ± 9.88	12.81 ± 9.08	0.26^a^
Length of stay, days, median (IQR)	5 (4.00-8.25)	5 (4.00-7.00)	12 (4.75-18.00)	0.01^a^

^a^Mann-Whitney U or paired t test; ^b^Fisher exact test or likelihood ratio test; DKA = diabetic ketoacidosis; IQR = interquartile range; DM = diabetes mellitus; APACHE II = Acute Physiology and Chronic Health Evaluation; SOFA = sequential organ failure assessment; SD = standard deviation.

## DISCUSSION

We conducted a single-center prospective prognosis cohort study on all patients older than 12 years of age who were admitted to the emergency department with DKA, with the aim of describing the incidence, precipitating factors, laboratory characteristics and inpatient and long-term mortality. Few studies have evaluated the characteristics and long-term outcomes of adult DKA patients, and the two major studies performed in Brazil were limited by their study design (retrospective medical record review) or focused solely on a pediatric population.^11^^,^^12^

Other studies focused only on intensive care unit (ICU) patients.^13^ However, one previous large study on DKA admissions found wide variability in ICU use for DKA patients (range: 2.1-87.7%), and found that hospitals with greater numbers of DKA admissions were less likely to admit these patients to the ICU.^14^ Likewise, in our study, although 37 (71%) of the patients had severe DKA, less than half were transferred to an intensive care unit, and most patients were treated in the emergency department. This confirms the importance of studying DKA patients within the emergency department setting and not only in the ICU.

We found that DKA represented less than 1% of total admissions at our hospital. Nonetheless, that still represented about one case a week over the one-year period. About two thirds of the patients were type-1 diabetics, but almost 20% of the patients with DKA had type-2 diabetes. Therefore, emergency department ED physicians need to consider DKA as a DM complication among type-2 diabetes patients presenting with hyperglycemia. 

We identified infection and noncompliance with DM treatment as the predominant precipitants for DKA, as previously shown by other studies.^8^^,^^9^^,^^10^ Noncompliance has repeatedly been identified as an important and potentially avoidable precipitant across all age groups.^10^^,^^15^^,^^16^^,^^17^ Psychosocial factors seem to contribute to noncompliance, including lower socioeconomic status and self-reported treatment error (i.e. incorrect management of insulin during illness).^10^

Moreover, some patients with both infection and treatment noncompliance have been counted within the rates for both of these precipitants. Many studies have not found a clear cause for some DKA patients, probably because of their retrospective design. In our study, we found the precipitating cause for every DKA case, possibly because our review of hospital records involved a careful search for medical impressions of treatment non-adherence and we objectively asked patients about treatment compliance preceding DKA. This may have revealed the true high rate of treatment non-adherence in DKA cases, as done previously by Weinert.^11^

Overall, the immediate inpatient mortality among adults in the United Kingdom and United States is less than 1%. However, it may reach rates higher than 5% among the elderly and among patients with severe comorbid conditions.^2^ On the other hand, DKA mortality remains high in developing countries, such as Kenya (30%) and Libya (11.7%).^18^^,^^19^ The immediate inpatient mortality associated with DKA in the present study was 5.8%, and it can be argued that our mortality rate has yet not reached the low levels of developed countries. It can also be argued that resource constraints, lower access to intensive care, public health issues and socioeconomic factors may contribute to higher mortality rates in developing countries.^20^^,^^21^ In addition, the mortality rate among our patients rose progressively and, after the two-year follow-up period, approximately one in every five patients with DKA admitted to the emergency department during the data-gathering period of our study had died. Although APACHE II had previously been shown to be an important predictor of DKA-associated mortality,^22^ that was not demonstrated by our data. Indeed, we did not find any correlation between inpatient mortality and the variables analyzed. Nonetheless, older and type-2 diabetes patients were at higher risk of long-term mortality, as also were those who presented with infection. This can be partially explained by the fact that sepsis survivor patients remain at substantially increased risk of death after recovery.^21^

Interestingly, although the ADA severity classification did not show any association with mortality, higher SOFA score at admission was associated with long-term mortality. The SOFA score ultimately represents dysfunction of target systems and organs, and we can argue that assessment of SOFA scores for DKA patients in the emergency department not only is important but also is essential, since these scores seem to predict mortality more accurately. 

Hypoalbuminemia was also associated with total long-term mortality among our DKA patients. Low albumin levels had already been correlated with mortality risk among hospitalized patients, and our data conﬁrm the findings of previous studies that found associations between low serum albumin and worse long-term outcomes.^23^

Regardless of the explanation, our study, like previous studies, seems to suggest that DKA patients still have a higher mortality rate long after discharge.^13^ This seems to be especially so among older type-2 diabetic patients, those with more than one comorbidity and infection at presentation, those with higher SOFA score and those with lower serum albumin at admission. Overall, we would like to propose that DKA should be understood as a mortality marker in DM patients and that such patients should be followed more closely by a diabetes specialist, in order to attempt to improve treatment compliance and maybe prevent some of these deaths over the long term, even though this has not yet been shown through research.^13^

Furthermore, some DKA episodes among DM patients might be preventable, given that noncompliance with treatment is a major precipitating factor. The fact that noncompliance remains one of the most common precipitant factors for DKA patients admitted to the emergency department would imply that an important gap exists between research, treatment options and healthcare delivery. These patients could benefit from patient-centered strategies that focus on DM education and address patients’ barriers to treatment and access to diabetes care.^2^^,^^24^

## LIMITATIONS

Firstly, this was a non-controlled study with a relatively small sample size that had few mortality events. This was mainly because we used a convenience sample, thereby limiting the statistical power. However, the design of this study was prospective and therefore it was singular, among studies performed at emergency departments in Brazil and elsewhere, especially considering its long-term (two-year) follow-up. 

Secondly, this was a single-center study and the DKA patients were admitted to a public tertiary-level teaching hospital in a large urban area in Brazil. Accordingly, our results may have limited generalizability to other areas that have differing healthcare systems. 

The classification of diabetes as type 1 or type 2 was determined through previous evaluation by the patient’s physician and this was not verified in this study. Cross-checking of the DM classification could not be performed because laboratory tests of greater sophistication, such as c-peptide and type-1 diabetes autoantibodies, were not available in the emergency department. It therefore remains possible that some of the DM patients could actually have presented rarer types of diabetes, such as LADA or MODY.

## CONCLUSIONS

We conducted the first Brazilian prospective study on DKA patients in the emergency department setting, with long-term follow-up. DKA in adult patients presenting to this department was found to be relatively frequent in our hospital and was associated with both short-term and long-term risk of death. Noncompliance and infection were major precipitating factors for DKA among these patients, and survivors remained at risk of long-term mortality. Thus, we propose that DKA serves as a mortality marker for DM patients, especially for those who are older type-2 diabetics, with infection, and who present with high SOFA score and hypoalbuminemia at hospital admission. Interventions aimed at overcoming therapy compliance barriers and providing nutritional support might prevent avoidable cases and improve the long-term outcomes.
